# Machine learning for flow batteries: opportunities and challenges

**DOI:** 10.1039/d2sc00291d

**Published:** 2022-04-07

**Authors:** Tianyu Li, Changkun Zhang, Xianfeng Li

**Affiliations:** Division of Energy Storage, Dalian National Laboratory for Clean Energy (DNL), Dalian Institute of Chemical Physics, Chinese Academy of Sciences Zhongshan Road 457 Dalian 116023 China zhangchk17@dicp.ac.cn lixianfeng@dicp.ac.cn

## Abstract

With increased computational ability of modern computers, the rapid development of mathematical algorithms and the continuous establishment of material databases, artificial intelligence (AI) has shown tremendous potential in chemistry. Machine learning (ML), as one of the most important branches of AI, plays an important role in accelerating the discovery and design of key materials for flow batteries (FBs), and the optimization of FB systems. In this perspective, we first provide a fundamental understanding of the workflow of ML in FBs. Moreover, recent progress on applications of the state-of-art ML in both organic FBs and vanadium FBs are discussed. Finally, the challenges and future directions of ML research in FBs are proposed.

## Abstract 2

A fundamental workflow of ML in flow batteries and recent progress of the state-of-art ML applications in both organic FBs and vanadium FBs are discussed. The challenges and future directions of ML research in FBs are proposed.

## Graphical abstract

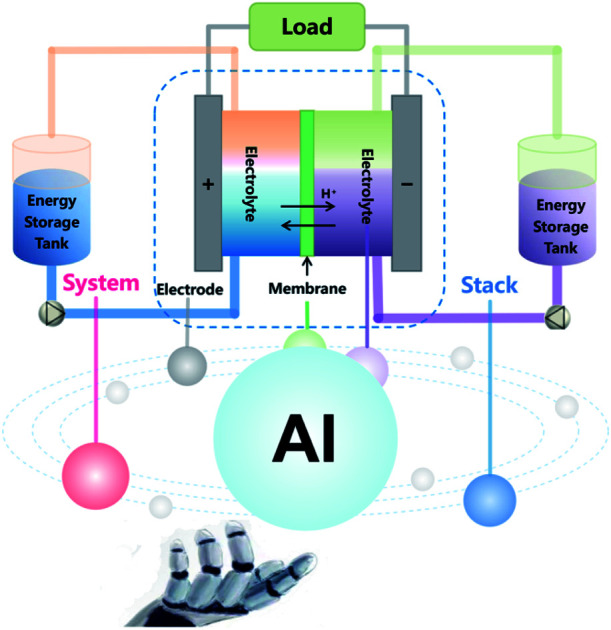

## Introduction

Worldwide economic growth has boosted the demand for energy, while the massive use of fossil fuels continues to cause environmental issues. To reduce greenhouse gas emission and meet the growing demand for energy consumption, more attention has been paid to renewable energy sources such as solar and wind power. To integrate the intermittent and unstable renewable energies into the grid, there is an urgent need for a safe, economic and environmental-friendly large scale energy-storage system to balance the renewable energy supply and electricity demand.^1–3^ Several energy-storage technologies, such as physical storage methods (*e.g.*, pumped storage hydro,^4^ flywheels,^5^ and compressed-air^6^), electrochemical methods (*e.g.*, Li-ion,^7^ lead-acid,^8^ and FBs^9,10^), and chemical methods (*e.g.*, hydrogen energy storage^11^), are available for electricity storage. Owing to the merits of decoupled energy and power, high safety, high efficiency, and long cycle life, FBs are well suited for the integration of renewable energy into the grid up to 100 MW for long term energy storage of 4 hours or longer.^10^

**Fig. 1:**
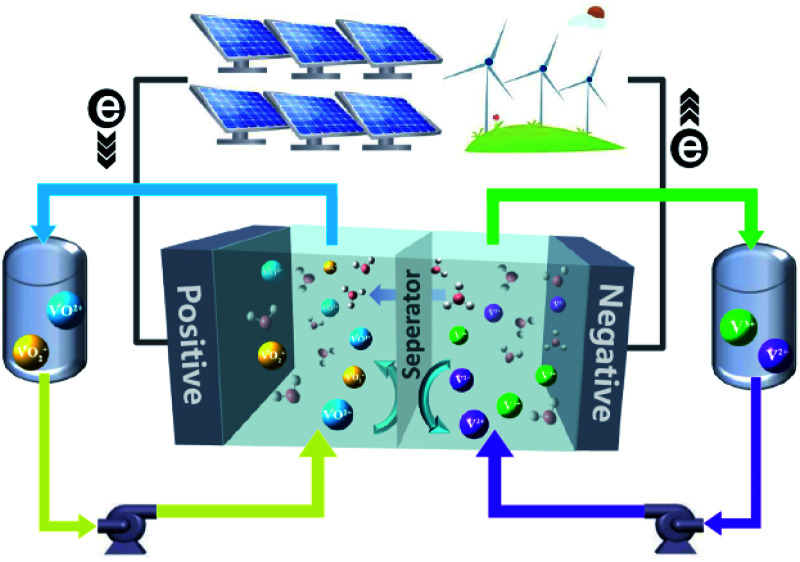
A schematic diagram of a VFB.

In a FB system, energy is typically stored in electrolyte solutions, which normally consist of redox-active couples (*i.e.*, catholytes and anolytes) and are separated on the opposite side of an ion conductive membrane. A schematic diagram of a vanadium flow battery (VFB) is provided in Fig. 1. Electrochemical redox reactions occur on the electrode surfaces in the electrochemical cell to convert chemical energy into electricity during discharge to supply the power, and the reversible electrochemical redox reactions do the opposite to store electricity during charge. The modern concept of FBs was proposed by the National Aeronautics and Space Administration (NASA) in 1970s,^12^ in which Fe^3+^/Fe^2+^ and Cr^3+^/Cr^2+^ redox-active couples were employed as the catholyte and anolyte, respectively.^13^ Since then, many new FBs have been designed, including metal- or inorganic-based redox species (*e.g.*, VFBs,^14,15^ Zn–Fe,^16,17^ Zn–Br,^18,19^ Zn–I,^20,21^ Zn–Mn^22^) and organic redox-active molecules (*e.g.*, quinones,^23,24^ TEMPO,^25,26^ viologen,^27^ and phenazine^25^). VFBs, which employ vanadium with different valences as catholytes (V^3+^/V^2+^) and anolytes (VO_2_^+^/VO^2+^), are regarded as one of the most promising large-scale energy storage technologies among the mental-based FBs, and have already been commercially implemented in recent years.^2^ Zinc-based FBs are another promising large-scale energy storage technology, owing to the low redox potential of −0.76 V (*vs.* the standard hydrogen electrode SHE) of the zinc deposition/stripping reaction, high theoretical capacity (820 mA h g^−1^, 5855 mA h cm^−3^), and low cost of zinc. However, the stability of zinc-based FBs needs to be further improved. Since the first organic FB system was reported,^23^ more research studies have focused on the discovery and design of novel organic redox-active species (ORASs). ORASs are normally composed of carbon, hydrogen, oxygen, nitrogen, and sulphur, which are earth-abundant elements.^9,28,29^ Thus, they could potentially offer low-cost electrolytes. Furthermore, the properties, including solubility, redox potential, kinetics, and stability, can be precisely tuned by molecular engineering.^30^ Therefore, organic flow batteries (OFBs) are regarded as a potential option for large-scale energy storage.

However, it is time-consuming to use traditional trial-and-error methods from the lab design to commercialization for a new material. Compared with traditional trial-and-error methods, computational chemistry has made great contributions to provide useful information for the research and development (R&D) of new materials, especially the density functional theory (DFT). Moreover, with the help of some modern chemical-simulation toolkits, the high-throughput calculation screening can speed up the discovery of new materials. However, the large-scale screening of new materials still takes too much time due to the high computational cost of high-precision DFT calculations.^31^

With the rapid development of computer ability and data science, AI (defined as the simulation of human intelligence processes by computer systems) has opened another door to modern research and attracted worldwide attention. In recent years, AI has been employed in the fields of natural language processing,^32^ image recognition,^33^ and autonomous driving.^34^ It shows great potential to surpass the existing cognitive level of human beings in some fields.^35^ ML is one of the most important independent disciplines of AI, which has been rapidly developed and widely applied recently.^36–38^ ML algorithms can automatically mine implicit relationships hidden behind the data from a large amount of data. With the development of chemoinformatics, it has vast applications in the fields of chemistry and material science, such as quantum chemistry,^39,40^ drug discovery,^41^ and molecular design,^42^ reaction prediction and reverse synthesis,^43^ and automated synthesis.^44^

In this perspective, we introduce the basic workflow of ML and discuss the R&D of ML in FBs, highlighting the successful applications of state-of-art ML in OFBs and VFB systems. Moreover, the prediction of the physical and electrochemical properties of organic redox-active molecules for FBs based on high-throughput computational simulations is included. Finally, we provide a perspective on the main limitations and future research directions of ML for FBs, and present a realistic outlook.

## The workflow of ML

**Fig. 2:**
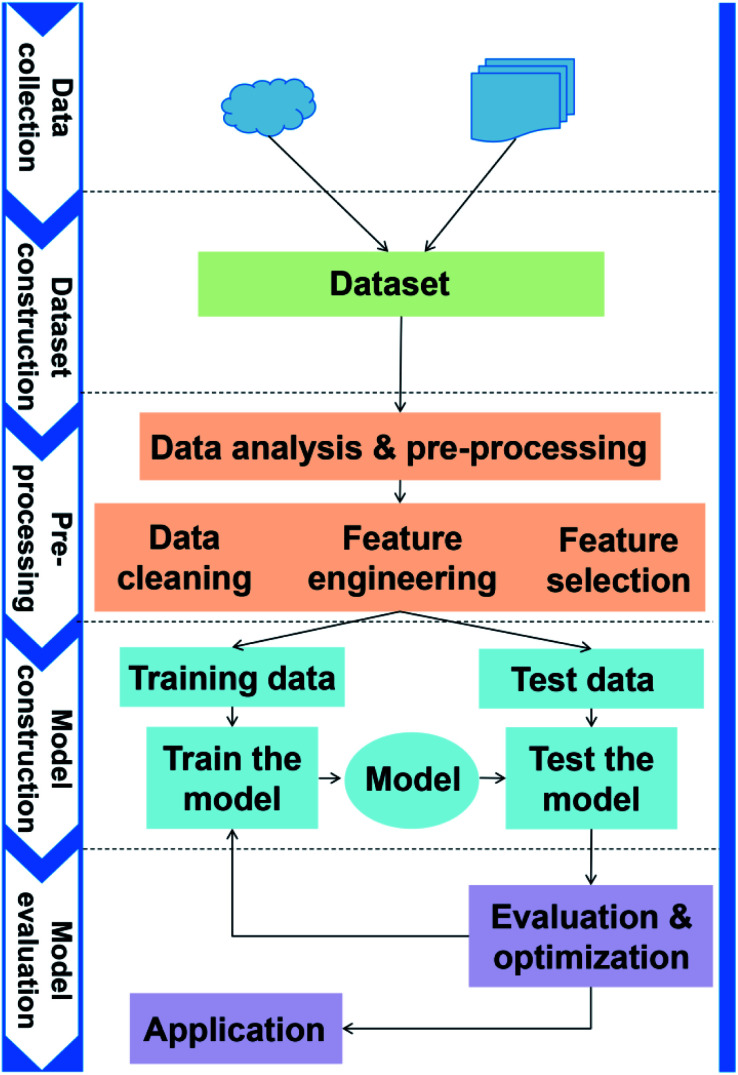
The general application workflow of ML.

The general application workflow of ML is shown in Fig. 2. ML is an interdisciplinary multi-field, involving probability theory, statistics, approximation theory, convex analysis, algorithm and others. It applies algorithms to establish the hidden relationships between massive data and specific properties. Thus, the first step is dataset construction, which collects sufficient data samples for ML application. The second step is feature engineering, which creates new features based on the raw data by mathematical representation. This mathematical representation should refine the correlation between features, resulting in a few new features to reflect the sample information. Followed by randomly dividing the data into training and test datasets, a ML algorithm is employed to establish a model between features and target function with training dataset. The parameters of the algorithm are adapted to achieve the goal that the accuracy and computation cost of the model are acceptable. Then, the accuracy (performance) of the model is evaluated by a test dataset. Finally, the validated model can be employed to predict the properties of unknown data.

### High-throughput calculations and database

A database is the basis of ML. Generally, the database can be constructed by the following methods.

#### (1) Doing high-throughput experiments or calculations

Severson *et al.* generated a database with 124 commercial lithium iron phosphate/graphite cells cycled under fast-charging conditions by experiment, and then applied ML to predict the cycle life of the cell by the discharge voltage curves from the early cycle capacity degradation.^45^

#### (2) Extracting and summarizing the data from publications and patents

However, most of the data reported in the literature are the best results under optimal conditions from successful experiments. The failed data from experiments are also generally deliberately hidden, which would cause serious data bias.

#### (3) Taking advantage of the existing open chemical or material databases

Such as GDB-13,^46^ GDB-17,^47^ ZINC,^48^ NREL materials database,^49^ OQMD^50^ and others. Some notable material databases are shown in Table 1. The computation method plays an important role in generating material properties. By employing open databases, we can avoid the time-consuming data preparation and generation process. Another advantage of databases obtained by high-throughput calculations is that they provide an open source and user-friendly Materials Application Programming Interfaces (API) for users.^51^ If the database does not include the properties we need, high-throughput calculations can be applied to obtain them. Some existing open-source automated interfaces, such as Materials Project,^52^ Python Materials Genomics (pymatgen),^53^ the open quantum materials database (OQMD)^50^ and others, can provide modules to perform high-throughput calculations including classical molecular dynamics and quantum mechanics calculations (*e.g.*, *ab initio* and DFT calculations).

**Table 1:** Some open source materials databases

Database name	URL	Descriptions
The Materials Project	https://materialsproject.org	Computed information on known and predicted materials including inorganic compounds, organic molecules, nanoporous materials
OMDB	https://omdb.mathub.io	An open access electronic structure database for 3-dimensional organic crystals
NRELMatDB	https://materials.nrel.gov	A computational materials database focus on materials for renewable energy applications
OQMD	https://oqmd.org	DFT calculated thermodynamic and structural properties of 815 654 materials
GDB-13	https://www.cbligand.org/gdb13	Databases of 970 million hypothetical small organic molecule
GDB-17	https://gdb.unibe.ch/downloads	Databases of 166 billion hypothetical small organic molecules
PubChem	https://pubchem.ncbi.nlm.nih.gov	Include freely accessible chemical information for small organic molecules
ZNIC	https://zinc.docking.org	A database for purchasable compounds
NIST Chemistry WebBook	https://webbook.nist.gov/chemistry	Thermochemical data for over 7000 organic and small inorganic compounds, reaction thermochemistry data for over 8000 reactions, IR spectra for over 16 000 compounds, mass spectra for over 33 000 compounds and so on
CCDC	https://ccdc.cam.ac.uk	A database for crystal structure data
COD	https://www.crystallography.net/cod	A database for crystal structures of organic, inorganic, metal–organics compounds and minerals, excluding biopolymers
ChemSpider	https://www.chemspider.com	Chemical information based on chemical structures, including physical and chemical properties of compounds

### Feature engineering

Feature engineering normally includes data pre-processing, feature extraction, selection and construction, which employs relevant knowledge in the data science to create features that enable ML algorithms to achieve the best performance. After the original dataset is constructed, feature engineering is a key step for applying ML algorithms. For an organic energy storage material, how molecules are represented (which is called a descriptor) and whether the descriptor contains key information will directly determine the performance upper limit of the model. The commonly used molecular descriptors include physicochemistry properties (*e.g.*, log *P*, p*K*_a_, molecular weight, and properties from DFT or semi-empirical calculations), molecular fingerprints (*e.g.*, MACCS keys,^54^ PubChem fingerprints,^55^ MolPrint2D,^56,57^ Morgan fingerprint,^58^ and others), molecular abbreviation (*e.g.*, SMILES,^59^ InChI,^60^ and SMARTS^61^), molecular graph (*e.g.*, Coulomb matrix^39^), and grid representation. After transforming a molecule into a computer-recognized mathematical representation, a feature selection method is employed to eliminate irrelevant or redundant features, so as to reduce the number of features, improve the model accuracy, and reduce the training time of the model. Filter feature selection, wrapper feature selection, and embedded feature selection are three commonly used methods. In short, feature engineering is a complex but extraordinarily crucial process for the application of ML, which directly affects the performance upper limit of the model.

### ML algorithms and models

Algorithms are the key to ML, and it can be generally divided into classical ML algorithms based on statistics and neural network. The classical ML algorithms normally include Bayesian, Decision Tree (DT), Support Vector Machine (SVM), Cluster analysis and Random Forest (RF). The Scikit-learn package^62^ in Python has integrated most of the classical ML algorithms, which can be easily accessed. The popular artificial neural network mainly includes fully connected neural network (FCNN), convolutional neural network (CNN), recurrent neural network (RNN), auto encoder (AE), and generative adversarial network (GAN). Many open source ML frameworks, such as TensorFlow,^63^ PyTorch,^64^ and Theano,^65^ can be accessed to establish the neural network.

Supervised learning algorithms are commonly applied in properties prediction of organic redox-active molecules. It requires labels (*e.g.*, physicochemical properties to be predicted) of the sample in the dataset. A higher accuracy of the learning model can be acquired with higher accuracy of the label and better representation of the sample. In specific applications, the most suitable ML algorithm can often be determined by screening and verifying existing algorithms. It should be noted that the optimal model based on the training dataset and the validation dataset may not be the most suitable model. This is due to the over-fitting or poor generalization ability of the algorithm. Therefore, it needs to be further tested and verified by an external test dataset to determine the optimal prediction model.

#### Linear model

Linear model is the simplest ML algorithm. Assuming that there are *n* samples, each sample includes a input vector ***X***_*i*_ = (*x*_1_^*i*^, *x*_2_^*i*^, …, *x*_d_^*i*^) and a corresponding value *y*_*i*_. In linear mode, the predicted value, *f*(***X***_*i*_) = *ω*_1_*x*_1_^*i*^ + *ω*_2_*x*_2_^*i*^ + … + *ω*_d_*x*_d_^*i*^ + *b* or *f*(***X***_*i*_) = ***ω***^*T*^***x***^*i*^ + *b*, where ***ω*** = (*ω*_1_, *ω*_1_,…, *ω*_d_) is the regression coefficients vector. The error between the difference of the predicted value *f*(***X***_*i*_) and the true value *y*_*i*_ is called loss. A loss function is the sum of each error for *n* samples. The mean squared error (MSE) is commonly used to avoid the positive and negative differences between the predicted value *f*(***X***_*i*_) and the true value *y*_*i*_. If we use MSE as the loss function, it can be calculated as follows,2.1
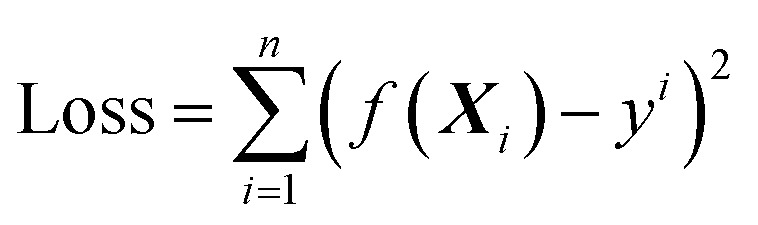


The object of the learning algorithm is to find optimal regression coefficients vector **ω** and intercept *b* to minimize the loss function. To prevent overfitting, regularization is normally used. Lasso regularization^66^ and ridge regularization^67^ are commonly used methods to prevent overfitting.

#### SVM

SVM is a supervised ML algorithm that can be used for both classification and regression. In SVM, each data item is plotted as a point in *n*-dimensional space (where *n* is a number of features) with the value of each feature being the value of a particular coordinate. The aim of the algorithm is to find the hyper-plane that differentiates the classes very well. For a regression problem, Support Vector Regression (SVR) is applied. Different from the aim of linear regression, which is to minimize the loss function, SVR gives the flexibility to define how much error is acceptable in the model and will find an appropriate line (or hyperplane in higher dimensions) to fit the data. There are five commonly used kernels functions, including linear kernel, polynomial kernel, Laplacian kernel, Gaussian kernel, and sigmoid kernel. SVM can solve high-dimensional problems with small samples, and can handle the interaction of nonlinear features without relying on the entire data set. However, the efficiency becomes poor if the samples are too large. The interpretation of high-dimensional features is not very clear, and it is sensitive to missing data and the kernel.

#### DT

DT is a supervised learning algorithm. A DT is a flowchart-like structure in which each internal node represents a “test” on an attribute, each branch represents the outcome of the test, and each leaf node represents a class label. There are three commonly used approaches for DT, which are ID3, C4.5, and CART algorithm. Random forest (RF) algorithm, which is a type of ensemble learning containing multiple DT models, can increase the robustness of a tree-based algorithm.

#### NN

Inspired by the action mechanism of neurons in the brain, a NN is composed of many nodes (called artificial neurons), containing an input layer, one or more hidden layers, and an output layer. Each node connects to another, and has an associated weight and threshold. If the output of any individual node is above the specified threshold value, that node is activated, sending data to the next layer of the network. Otherwise, no data is passed along to the next layer of the network. Based on different purposes, NNs can be classified into different types, including perceptron, deep neural network (DNN), convolutional neural network (CNN), recurrent neural network (RNN) and others. The activation function is one of the important parts in NNs. The input is calculated by the active function in the neural network, and the result is passed to the next neuron or output as the result of the NN model. Optimization algorithms, such as stochastic gradient descent (SGD), RMSprop, Adagrad, Adadelta and Adam, are commonly used to calculate and optimize the loss of the model.

### Model evaluation

Evaluating the model is a core part of building an effective ML model. The ML model learned by the algorithm cannot cover all situations, which will lead to a difference between the actual predicted output and the true value of the sample. The error of the ML model on the training and test set is called the training error and test error, respectively, and the error on the new sample is called the generalization error. The aim of the ML model is to have a small generalization error. However, it is hard to calculate the generalization error. Thus, the commonly used method is to divide the dataset into a training set, a validation set and a test set, and they are applied to train the model, adjust the parameters, and calculate the test error, respectively. The test error is regarded as the approximate evaluation of the generalization error to evaluate the accuracy of the ML model.

#### Bias and variance

The generalization error is the sum of bias, variance and noise, which is determined by the learning algorithm, the sufficiency of the data, and the difficulty of the learning task, respectively. If the bias is small but the variance is high, the ML model is overfitting, which indicates the model is too complex. If the bias and variance are both very high, the ML model is underfitting, which indicates the model is too simple. The process to balance the overfitting and underfitting is to adjust the internal variables (hyperparameters) of the model. Cross validation can be applied to evaluate the generalization performance of the model, which is commonly used in ML.

## Application of ML in FBs

In this section, the application of ML for the design and development of organic redox-active species (ORASs) and VFBs is summarized. Database, which was built through high-throughput virtual screen (HTVS) for ORASs is first introduced. The application of ML mainly focuses on redox potential, solubility and stability prediction. Thus, we discuss the establishment of the model for each specific problem and the performance of the ML model. Finally, some ML applications in VFBs, such as electrode structure, membrane optimization and system cost and performance prediction, are reviewed.

### Database construction for ORASs

**Fig. 3:**
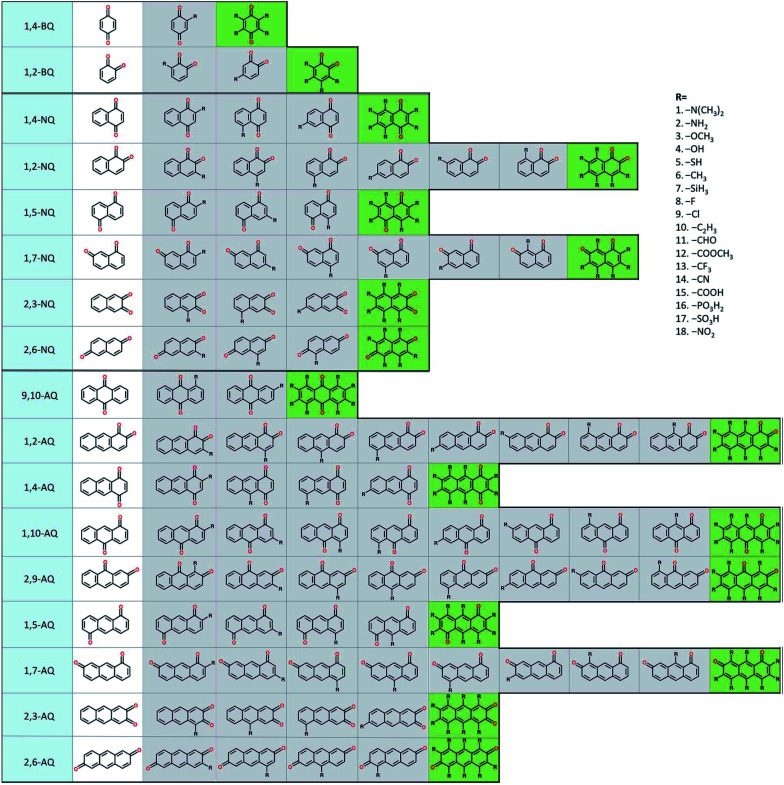
A schematic representation of the molecular screening library. The parent BQ, NQ, and AQ isomers are shown on the left (white). These quinone isomers are functionalized with 18 different R-groups singly (gray) and fully (green) to generate a total of 1710 quinone molecules. Reproduced with permission from ref. 68. Copyright 2015 Royal Society of Chemistry.

To build the structure–property relationship for ORASs, a database is needed. HTVS have been found to be an effective technology for database generation, especially for organic molecules, owing to its low cost and high efficiency. Er *et al.* has applied a HTVS approach to study potential candidate organic molecules for quinone derivatives using DFT calculations.^68^ The virtual library includes 17 core structures of quinones, which were decorated with 18 substituents (–N(CH_3_)_2_, –NH_2_, –OCH_3_, –OH, –SH, –CH_3_, –SiH_3_, –F, –Cl, –C_2_H_3_, –CHO, –COOCH_3_, –CF_3_, –CN, –COOH, –PO_3_H_2_, –SO_3_H, and–NO_2_), as shown in Fig. 3. The single and full substitutions were taken into consideration for the redox potential and solvation-free energy calculation. DFT calculations were employed to calculate the energy of the quinone molecules. The total database includes 1710 quinone (Q)/hydroquinone (QH_2_) molecular couples. Results showed that it should be possible to adjust the standard redox potential from as high as 0.6 V_NHE_ to −1.5 V_NHE_. Ultimately, 408 Q/QH_2_ couples were screened with *E*^0^ < 0.2 V *vs.* SHE and *E*^0^ > 0.9 V *vs.* SHE. Tabor *et al.* extended the research of the redox potential and solvation-free energy to stability for Q/QH_2_ couples.^69^ They combined DFT and semi-empirical calculations to study the decomposition or instability mechanisms from the virtual screening of more than 140 000 quinone pairs. Their research indicated that HTVS is an effective method to construct databases for ORASs.

**Fig. 4:**
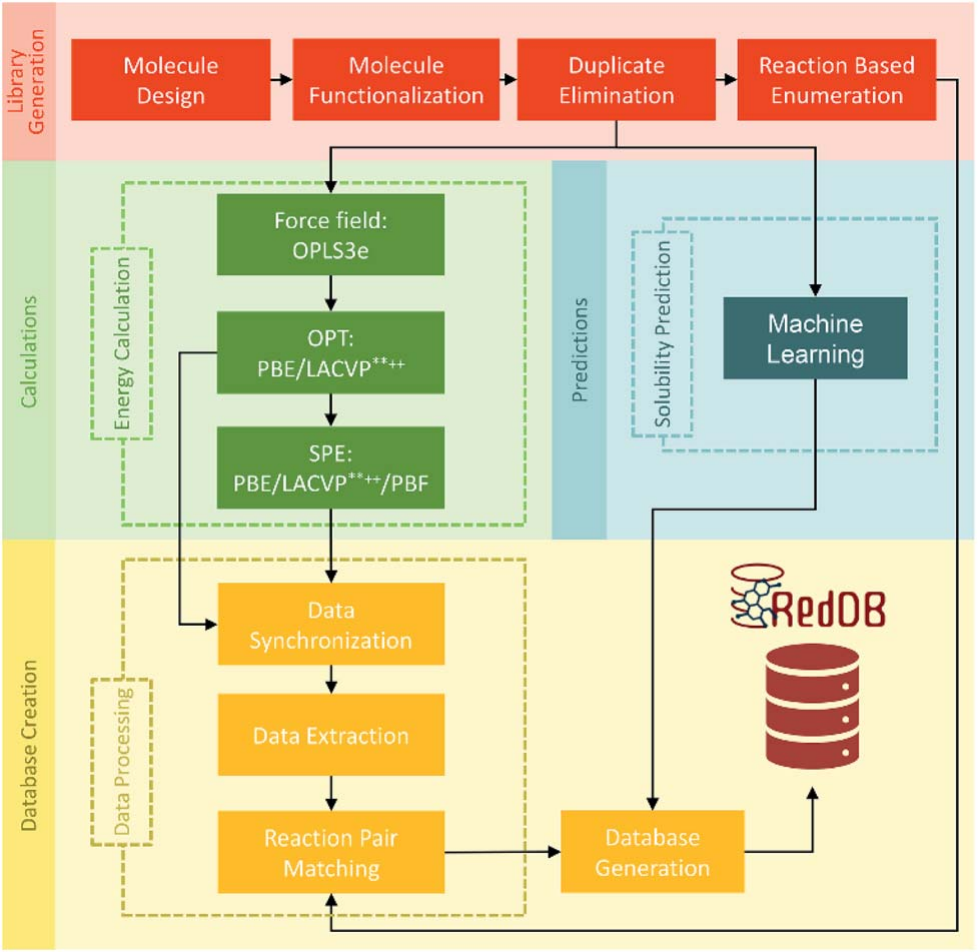
A schematic overview of the various tasks that have been undertaken for the development of RedDB. Reproduced with permission from ref. 71. Copyright 2021 ChemRxiv.

Carbonyl reductions to alcohols and amines are two of the most common carbon redox transformations in biology. The database of redox potentials of carbonyl reductions to alcohols and amines can also be used for ORASs. A constructed database with standard potentials of more than 315 000 redox reactions involving approximately 70 000 compounds by PM7 calculation was calibrated with Gaussian process (GP) regression, which can be fully assessed at https://github.com/aspuru-guzik-group/gp_redox_rxn.^70^ The construction of the molecular structure–property database using the HTVS strategy for ORASs mainly includes three stages: molecule library generation, molecule structure and property generation, and database creation.^71^ The process for the development of RedDB is shown in Fig. 4.

The main weakness of HTVS is the failure to address the error between the calculated value and the experiment. Wedege *et al.* systematically studied the redox potential, solubility, and stability of 28 quinone-based compounds by experiment, and found that the position of the substituents plays a key role in determining the redox potential and solubility, while the number of substitutions only has a minor influence.^72^ Their experimental data showed that the standard redox potential of sulfonated AQs [electron-withdrawing groups (EWGs)] is only 200–300 mV higher than that of hydroxylated ones [electron-donating groups (EDGs)], which was not fully consistent with the EWGs increasing the redox potential of the anthraquinone (AQ) derivatives, while EDGs decrease the redox potential of AQs.^68^ The reliability of each sample in the database is the basis of ML. Therefore, the accuracy of theory calculation for HTVS needs to be verified in the first place. Another weakness of HTVS in building a database is that the calculation level employed by researchers varies from one to another, which builds a barrier for other researchers when combining these databases for further ML applications. Thus, more comprehensive, standard data samples should be established to share between databases.

### Applications of ML in ORASs

ML is widely applied in the field of property predictions. For ORASs, the solubility, redox potential and the stability are most frequently involved. Ideal ORASs should have high solubility, rational redox potential and high stability to have high energy density and long cycle life. However, obtaining these properties by experiments and high-precise computation is expensive and time-consuming. Therefore, the ML approach with high efficiency attracted much attention for these property predictions. Since the accuracy of the ML model is highly related to the amount and reliability of the data, the prediction of the properties of ORASs is often combined with high-throughput quantum mechanical calculations.

**Fig. 5:**
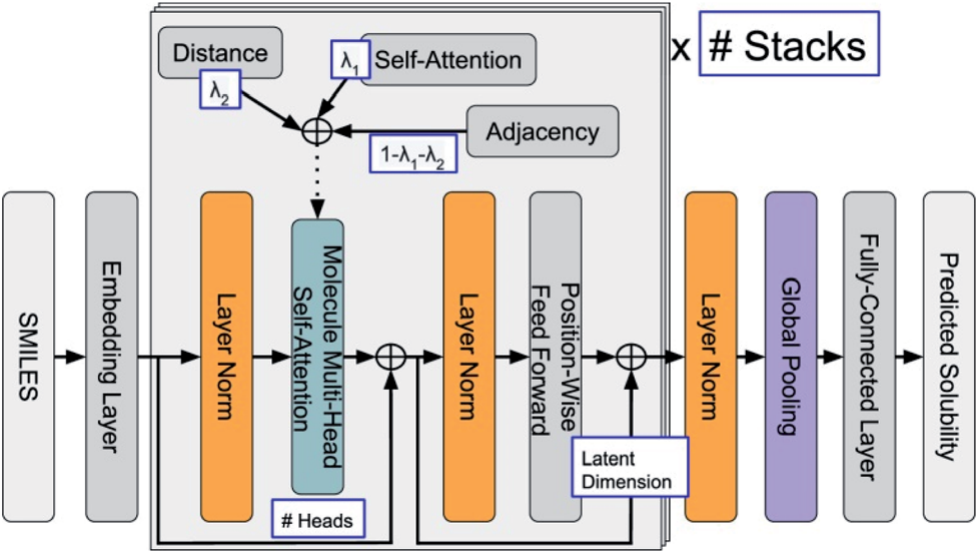
General architecture of SolTranNet. Each item in a blue box is a tuned hyper-parameter. Reproduced with permission from ref. 82. Copyright 2021 American Chemical Society.

The estimation of aqueous solubility of organic compound has been widely studied for many years.^73–82^ The prediction methods can normally fall into three categories: the first type is based on group contribution method.^73,83^ The second employs regression from molecular parameters, which is also regarded as quantitative structure–activity relationships (QSAR).^84,85^ The third applies a data-driven approach (ML algorithms), including SVM,^75,77^ RF,^86^ NN,^80,87^ and others,^81^ to establish a correlation between the molecular structure and solubility. In 2019, Sorkun *et al.* built a new database AqSolDB, which consisted of 9982 unique organic compounds, for the prediction of aqueous solubility.^88^ In 2021, Francoeur *et al.* proposed a ML tool, SolTranNet (the general architecture is shown in Fig. 5) which contained 3393 parameters, for the fast aqueous solubility prediction based on the AqSolDB dataset.^82^ A specific ML model called multiple descriptor multiple kernel (MultiDK) method was developed to discover ORASs for aqueous organic flow batteries (AOFBs).^89^ By combination of binary descriptors (*e.g.*, Morgan fingerprint and the MACCS keys) and nonbinary descriptors (*e.g.*, the physicochemical molecular property), the prediction accuracy of aqueous solubility improved significantly. The combination of the Tanimoto similarity kernel for binary descriptors and linear kernel for nonbinary descriptors showed better solubility prediction performance than LR. As a result, MultiDK can predict pH-dependent solubility for quinone and its derivatives. Although many solubility models have been developed and validated by researchers, the overall performance of these models for a blind dataset was not as encouraging as that reported in the published papers.^90^ The main reason is the lack of high quality experimental data and structural diversity of the training dataset. Thus, to construct more reliable solubility prediction models, more attention was needed to obtain high quality experimental data and a diverse molecular structure dataset construction.

**Fig. 6:**
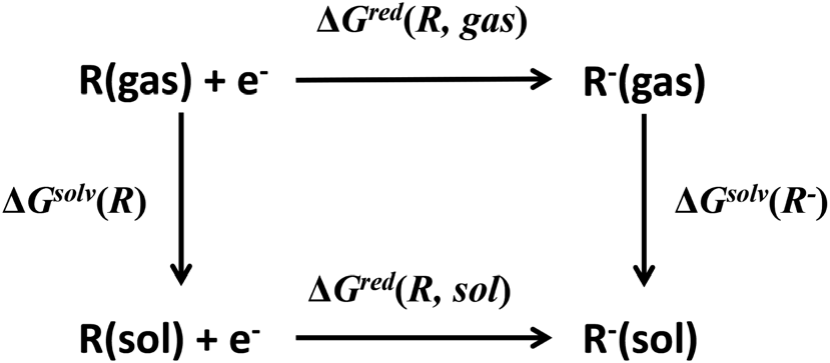
Thermodynamic cycle to calculate the equilibrium redox potential in the solution. Reproduced with permission from ref. 93. Copyright 2020 Elsevier Ltd. All rights reserved.

Redox potential is another important property for ORASs. The redox potential of an organic redox-active couple can be calculated from the thermodynamic cycle (Fig. 6) by first-principles computational methods,^91^*e.g.*, DFT calculation.^92^ Recently, some structure-redox potential prediction models were established by ML to predict the redox potential of ORASs.^70,93–96^ For instance, Doan *et al.* applied the Gaussian process regression (GPR) to forecast the oxidation potential of homobenzylic ethers (HBEs) molecules for application in nonaqueous flow batteries.^97^ The dataset was constructed by DFT calculations, and contained 1400 HBEs. The GPR model contained a Matérn kernel, a generalization of the radial basis function, to construct the covariance matrices. A total of 9% of HBE molecules were identified with the desirable *E*^ox^ ∈ [1.40 V, 1.70 V, *vs.* NHE]. An active learning framework based on Bayesian Optimization (BO) was then applied to find materials with desirable oxidation potentials more efficiently. Their results showed that the BO were more than 5-fold improvement in computational efficiency compared to the random selection.

**Fig. 7:**
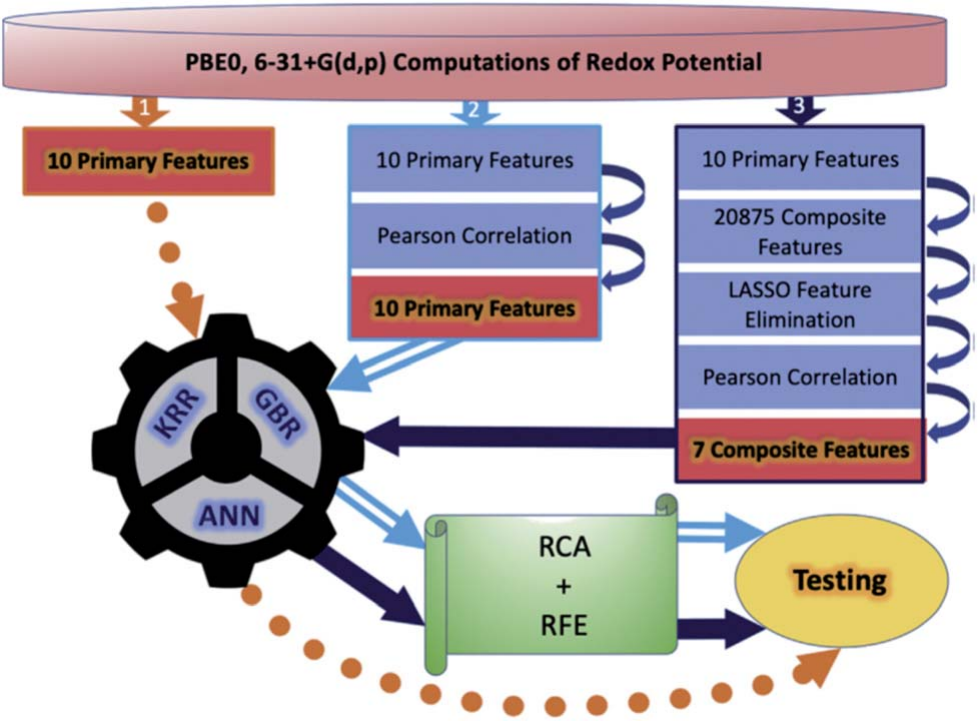
Overall breakdown of the three pipelines for all three learning models. Pipeline 1 represents the base protocol, in which the models were trained directly using the 10 primary features. *Pipeline 2* depicts the placement of a Pearson correlation filter, in addition to a relative contribution analysis (RCA) and recursive feature elimination (RFE). Lastly, *pipeline 3* depicts the addition of composite features and feature elimination using LASSO. Reproduced with permission from ref. 93. Copyright 2020 Elsevier Ltd.

Although a practical application example of ML to discover novel ORASs directly and then synthesize accordingly has been rare up to now, ML still provides a meaningful future vision for accelerating the discovery of new battery materials. Most recently, by combining ML with high-precision theoretical calculations and experiments, Zhang *et al.* found that indigo trisulfonate [Indigo-3(SO_3_H)] showed higher solubility, capacity retention, and coulombic efficiency than AQDS and its predecessors.^96^ Allam *et al.* applied high-throughput screening methods based on the DFT-ML framework to design novel organic electrode materials of Li-ion batteries.^93,98^ Their dataset includes various derivatives of functionalized graphene flakes, ketones, quinones, corannulenes, and coronenes, which can be found from their previous work.^99–103^ DFT was employed to calculate the primary feature (electronic properties) for the input of ML, including electron affinity, highest occupied molecular orbital (HOMO) and lowest unoccupied molecular orbital (LUMO), and the HOMO–LUMO gap. Three pipelines were used for each of the three algorithms, including ANN, gradient boosting regression (GBR), and Kernel ridge regression (KRR), to train the models, which are summarized in Fig. 7. The results showed that the model trained by KRR in *Pipeline3* has the best performance (with an MSE of 0.025) for the prediction of the redox potential. The trained KRR model was tested by an unknown dataset, which includes 17 sumanene derivatives. An average error and a Pearson correlation of 3.94% and ∼97%, respectively, were obtained between the DFT- and KRR-predicted redox potentials.

**Fig. 8:**
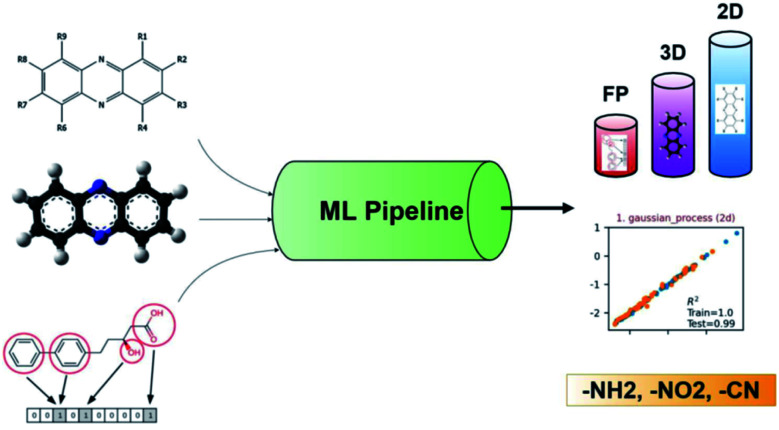
The pipeline of applying ML to predict the redox potentials of phenazine derivatives. Reproduced with permission from ref. 96. Copyright 2021 ChemRxiv.

Recently, ML was successfully employed in the prediction of the redox potentials of phenazine derivatives.^96^ The workflow is shown in Fig. 8. The dataset includes 185 molecules. 2D, 3D and molecular fingerprints were generated as the input features. Twenty linear or non-linear ML algorithms (LR, Ridge Regression, Lasso, Elastic-Net, LARS Lasso, Orthogonal Matching Pursuit, Bayesian Ridge Regression, Automatic Relevance Determination Regression, Passive Aggressive, Huber Regression, Kernel ridge Regression, SVM, GPR, Decision Trees, Bagging meta-estimator, Random Forest, AdaBoost, Gradient Boosting Regression, Artificial Neural Network, Nearest Neighbors Regression) were employed to build the redox potential prediction model, and high accuracy was obtained for all of the above models for both training and test datasets (*i.e.*, *R*_2_ > 0.98, MSE < 0.008 V and MAE < 0.07 V). Their results indicated that the linear model can obtain high performance when the features are properly selected. The trained best-performance ML model was applied to predict the redox potential of previously reported promising ORASs, including tetra-amino-phenazine (TAPZ), hexa-amino-phenazine (HAPZ), and octa-amino-phenazine (OAPZ), and the predicted redox potential was less than 0.07 V (<3%).

### Implementation of ML in FBs

**Fig. 9:**
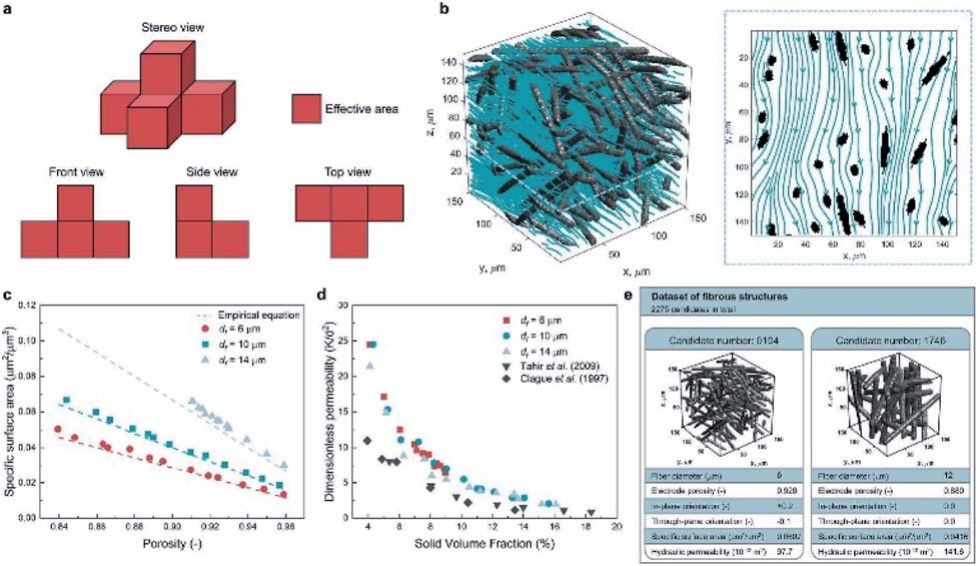
Computational methods used in the dataset generation. (a) A simple example illustrating the calculation method of the specific surface area. The area of voxel facets belonging to both the solid phase and pore phase is regarded as the effective area (colored in red). (b) Streamlined plot of the simulated velocity field within a three-dimensional fibrous structure. The insert is the streamlined plot of the slice *z* = 60 μm. (c) Comparison between the simulated specific surface area and the empirical equation (filament analogue model). (d) Comparison between the simulated hydraulic permeability. (e) Illustration of the two examples stored in our dataset. Each case has the four input variables and the two output variables. Reproduced with permission from ref. 104. Copyright 2021 Elsevier Ltd.

Apart from predicting the properties of ORASs, ML can also be utilized in electrode design,^38,104^ membrane design^105^ and system optimization for FBs.^106^ As the place in which electrochemical reaction occurs, the pore structure and specific surface area of the electrode will affect the efficiencies of the FB. Wan *et al.* combined a data generation method with ML algorithms to design porous electrodes with large specific surface area and high hydraulic permeability for FBs.^104^ Stochastic reconstruction method, morphological algorithm and lattice Boltzmann method were adopted to construct the dataset, which contains 2275 fibrous structures (shown in Fig. 9). LR, ANN, and RF algorithms were employed to construct the model for the specific surface area and hydraulic permeability prediction of porous electrodes. ANN achieved the best accuracy with a test error of 1.91% and 11.48% for specific surface area and hydraulic permeability, respectively. More than 700 promising porous electrode materials were screened by combination of a genetic algorithm with ANN. Graphite felt electrodes with an 80% increase for the specific surface area and 50% increase for the hydraulic permeability compared to the commercial one can be found, which indicates that ML has an attractive potential in the design of porous electrode materials for FBs.

**Fig. 10:**
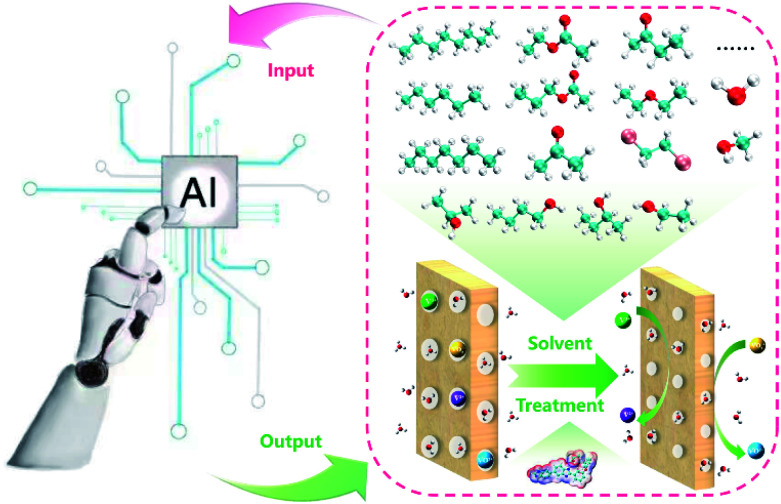
A schematic workflow of applying ML to screen suitable solvents for the solvent treatment of a PBI porous membrane. Reproduced with permission from ref. 105. Copyright 2021 Royal Society of Chemistry.

The membrane is one of the key components in VFBs. The performance of VFB is directly affected by membranes. Recently, LR and ANN algorithms were applied to predict the performance of a PBI porous membrane treated by various solvents (Fig. 10). Nine solvent properties and five experimental parameters were used as input.^105^ The mean absolute percentage error (MAPE) of the above models can be achieved within 1% for both voltage efficiency (VE) and energy efficiency (EE). The reliability of this model was further demonstrated by experiment. This model was further applied to screen for the proper solvent for the solvent treatment of the PBI porous membrane, and alcohols were regarded as the most suitable solvent to regulate the porous structure.

ML can be applied to optimize and design the microstructure of electrodes combined with the computational fluid dynamics simulations, and to guide the surface functionalization combined with DFT calculations. For membranes, ML can be applied to optimize the fabrication conditions and to understand the polymer (or porous) structure–property (*e.g.*, ion selectivity and ion conductivity) relationship. For electrolytes, ML shows enormous potential in ORASs design and the optimization of electrolyte interactions.

Currently, VFBs are at a commercial demonstration stage. However, the relatively high cost restricts their further commercialization. The performance and cost of a VFB system is highly related to the stack and electrolyte. Thus, it is extremely important for the optimization of VFB stacks and systems in a more efficient way. LR models were thus established to predict the power cost, energy cost, voltage efficiency (VE), energy efficiency (EE), utilization ratio of the electrolyte (UE) of VFB systems based on the operating current density, materials and structure parameters.^106^ The MAPEs of these models can achieve a precision within 1% for VE and EE, and within 5.2% for UE. The coefficients of the models demonstrated that the future development of materials for the VFB stack should focus on reducing the electrochemical polarization and ohmic polarization at high current densities, and the design of the flow field should monitor the enhancement of mass transfer to decrease the concentration polarization of FB stacks.

## Summary and prospects

**Fig. 11:**
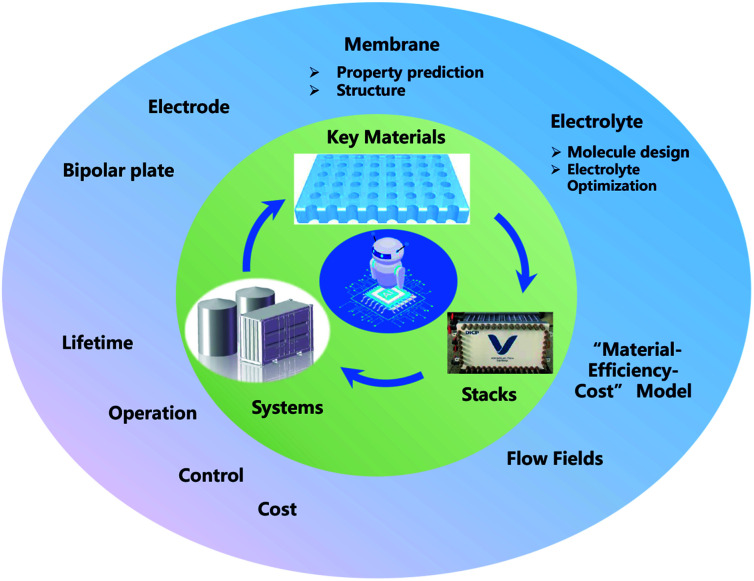
Prospects for the future research of ML for FBs.

ML, especially deep learning technology, has already been applied in the development of FBs, from key materials design to system performance–cost relationship optimization. However, in spite of these meaningful advances that have been achieved in the past decade, ML in FBs is still in its infancy. Thus, enormous work should be paid to establish effective ML models for FBs (Fig. 11).

First, a primary focus can be put on the construction and sharing of the relative database and algorithms. A database with the sufficient amount of data and reliable data is the first key step in the wide application of ML in FBs. How to share the data more efficiently is another challenge. Currently, there are only some open sources of chemoinformatics databases,^71,107^ and the validity and amount are limited. In addition, innovative ML algorithms, including clustering, principal component analysis (PCA), autoencoder (AE), generative adversarial network (GAN) and meta-learning models like neural Turing machines^108^ and imitation learning algorithms,^109^ are promising solutions for key materials design in FBs. Finally, the closed-loop design of key materials in FBs has not been formed yet. Future research should combine simulation, ML, and experiments to truly realize the effective guidance and application in material design.

Improving the performance of the stack and reducing the cost of the system are critical for further application of FBs. ML has been already applied to connect the stack performance and system cost of VFB.^98^ The performance-cost models can also be built for other FBs, such as Zn-based and organic FB systems. Moreover, more attention can be paid to the combination of the time series prediction methods, such as the ML algorithm with computational fluid dynamics (CFD) simulations, to establish the concentration gradient, electric field gradient, and pressure gradient models. This can further guide the design of the flow field structure and improve the performance of the stack.

Monitoring system operating parameters (such as voltage, current, state of charge (SOC), and temperature) and the further prediction of these parameters are critical to the stable and safe operation of FB systems. ML can also be applied to predict the above operating parameters based on the huge amount of operating data generated by the FB systems, and further guide the operation of the systems. Moreover, ML can be applied to optimize the overall cost of a FB system by establishing a model between the operation parameters and performance. With the establishment of more commercial FB systems, a large amount of data (*e.g.*, efficiencies, capacity, charge–discharge curve of each cycle) will be generated during the operation of those systems in the future. These data can be used by ML to establish the operational performance models of the FB systems (*e.g.*, battery cycle life^45,110^), which can be involved in the intelligent control system. Considering the high cost to build a FB system, the abovementioned research requires the cooperation between researchers and enterprises. If enough FB systems data are collected, ML can be applied to predict the cycle life and lower the system cost.

Interpretation of ML can provide inspiration and supplemental information for the understanding of mechanisms and laws for the original design, discovery of energy materials, and guide the optimization of stack and systems. Therefore, how to establish a model with a clear mechanism, which can be understood from a human perspective, will be an important research direction in the future and will mutually promote the novel key materials design of FBs.

## Data availability

Data sharing is not applicable to this article as no new data were created or analyzed in this study.

## Author contributions

Tianyu Li: investigation, visualization, funding acquisition and writing – original draft. Changkun Zhang: conceptualization, supervision and writing – review & editing. Xianfeng Li: conceptualization, funding acquisition, supervision and writing – review & editing.

## Conflicts of interest

There are no conflicts to declare.

## Supplementary Material
